# Standard Enucleation with Aluminium Oxide Implant (Bioceramic) Covered with Patient's Sclera

**DOI:** 10.1100/2012/481584

**Published:** 2012-04-30

**Authors:** Gian Luigi Zigiotti, Sonia Cavarretta, Mariachiara Morara, Sang Min Nam, Stefano Ranno, Francesco Pichi, Andrea Lembo, Stefano Lupo, Paolo Nucci, Alessandro Meduri

**Affiliations:** ^1^Ophthalmology Unit, Department of Surgical and Anesthesiological Sciences, S. Orsola-Malpighi Hospital, University of Bologna, 40137 Bologna, Italy; ^2^Ophthalmology Clinic, 00043 Ciampino, Italy; ^3^Ophthalmology Unit “Ciardella”, S. Orsola-Malpighi Hospital, 40137 Bologna, Italy; ^4^Department of Ophthalmology, CHA Bundang Medical Center, CHA University College of Medicine, Sungnam 463-760, Republic of Korea; ^5^University Eye Clinic, San Giuseppe Hospital, 20123 Milan, Italy; ^6^Department of Ophthalmology, University of Rome “Sapienza”, Polo Pontino, 04010 Latina, Italy; ^7^Ophthalmology Unit, University of Messina, 98100 Messina, Italy

## Abstract

*Purpose*. We describe in our study a modified standard enucleation, using sclera harvested from the enucleated eye to cover the prosthesis in order to insert a large porous implant and to reduce postoperative complication rates in a phthisis globe. *Methods*. We perform initially a standard enucleation. The porous implant (Bioceramic) is then covered only partially by the patient's sclera. The implant is inserted in the posterior Tenon's space with the scleral covering looking at front. All patients were followed at least for twelve months (average followup 16 months). *Results*. We performed nineteen primary procedures (19 patients, 19 eyes, *x* M; *x* F) and secondary, to fill the orbital cavity in patients already operated by standard evisceration (7 patients, 7 eyes). There were no cases of implant extrusion. The orbital volume was well reintegrated. *Conclusion*. Our procedure was safe and effective. All patients had a good cosmetic result after final prosthetic fitting and we also achieved good prothesis mobility.

## 1. Introduction

After enucleation and evisceration, the socket loss becomes another potential problem. Placement of an orbital implant can provide good cosmesis and proper motility.

Porous orbital implants have been popular in recent years, because they own several advantages over traditional alloplastic implants. 

The relatively light weight of porous implants may reduce pressure over the lower lids, decreasing the likelihood of lower eyelid sag and sulcus deformity [1].

Porous implants also permit the placement of a peg for direct coupling to a prosthesis, allowing better prosthesis motility, and supporting the weight of the prosthesis to reduce the risk of developing lower lid laxity with long-term prosthetic wear [2].

Less implant migration and extrusion are found because of better fibrovascular ingrowth of microporous structure [1, 2].

Numerous microporous orbital implants have been used to replace orbital volume including glass, ivory, coralline hydroxyapatite (HA) (Bio-Eye) [3], the porous polyethylene (Medpor) [4], and the aluminium oxide (Bioceramic) [5].

Risk factors for early dehiscence include previous surgery or trauma, radiotherapy, and secondary implantation [6]. When an orbital implant becomes exposed, it is often necessary to resort to further surgery [7]. 

Autogenous fascia, cadaveric sclera or fascia, bovine pericardium, or synthetic material such as polyglactin mesh have been used to cover an exposed hydroxyapatite orbital implant [7–10].

One of the drawbacks with a traditional evisceration is that there is a limit to the size of the implant that can be placed within the scleral cavity. With a standard evisceration, it is usually not possible to place anything larger than a 16 mm sphere [11]. Several modifications to the procedure have been reported to enable the placement of larger implants within the scleral cavity. Most of these techniques include cutting the sclera anteroposteriorly with or without separating the optic nerve from the scleral flaps [12–14].

We conducted a prospective interventional study assessing the efficacy of an alternative surgical technique that combine evisceration with enucleation procedure in order to insert a larger porous implant in a phthisis globe.

## 2. Materials and Methods

This study was conducted at the Eye Clinic of the University of Bologna, S. Orsola-Malpighi Hospital, Bologna, Italy and it was approved by the local Ethical Committee and adhered to the tenets of the Declaration of Helsinki.

26 consecutive patients who underwent surgery between January 2009 and December 2010 were enrolled in the study, 19 for a primary evisceration and 7 for secondary implantation.

The type of procedure and the implant used, the characteristics, and benefits of operation were explained to the patients and a written consent was obtained.

All patients signed informed consent forms for surgery. Surgeries were performed by one expert surgeon (G. L. Zigiotti).

Patients were all seen at 1 and 3 weeks and 3, 6, 12 months postoperatively by the medical staff. An assessment was made of the socket in all cases. Comment was passed on the conjunctival wound and implant motility, and the presence of conjunctival dehiscence was recorded.

### 2.1. Surgical Technique

**Figure 1:**
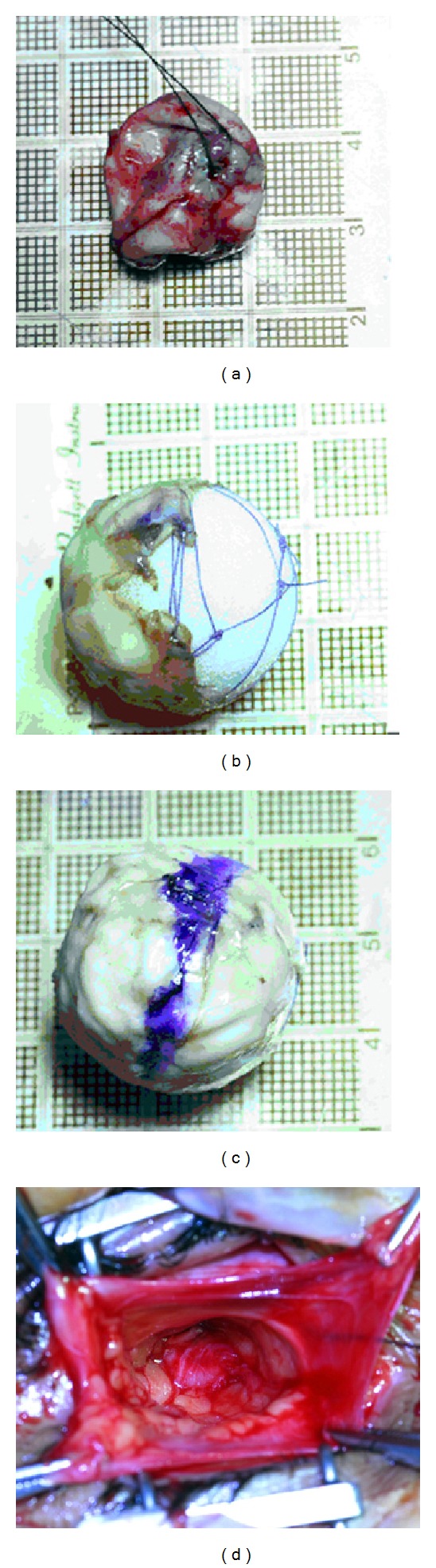
Phases of surgical preparation of the phthisis globe. (a) Enucleation of phthisis bulbous. A traction suture is placed on the cornea. (b) The sclera covers only the anterior surface of the porous implant; a mesh Vicryl suture maintains the sclera in a fixed position. (c) The sclera is longitudinally marked in blue color. (d) The posterior Tenon's fascia is dissected to receive the prothesis.

Since a traction suture was placed on the cornea, a 360-degree limbal peritomy was made. In order to secure the four rectus muscles, a suture was inserted at the end of each of them while the superior and inferior oblique muscles were cut off. A standard enucleation was performed and the optic nerve was transected (Figure 1(a)). The globe was prepared to receive the bioceramic implant using an accessory surgical table. The cornea and the intraocular uveal tissues contents were removed by means of an evisceration spoon. The corneal hole was enlarged by bilateral Z incisions in order to insert a 22 mm or 20 mm bioceramic prothesis. The sclera was placed in an antibiotic solution for five minutes.

**Figure 2:**
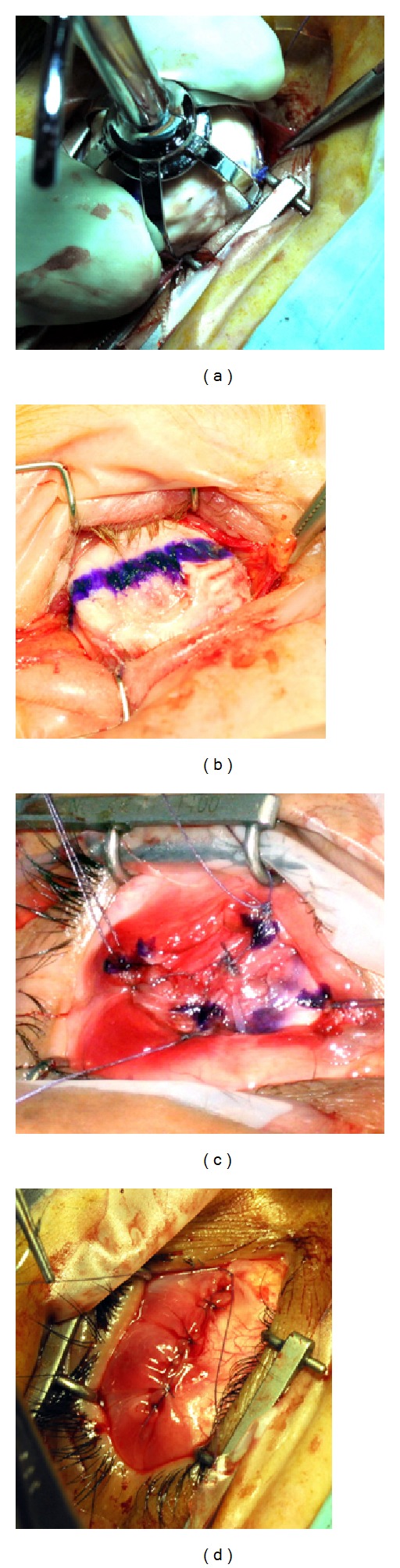
Insertion of the bioceramic prothesis. (a) The partially covered implant is inserted. (b) The marked pole is in front. (c) The four rectus muscles are sutured on the posterior Tenon's fascia. (d) The conjunctival layer of the suture is finished.

The 5-0 Vicryl single suture mesh implant was inserted and the sclera was sutured (Figure 1(b)). The posterior part of the sclera was marked by a surgical pen (Figure 1(c)). The hole corresponding to the optic nerve entrance was enlarged using a finger (Figure 1(d)), and the implant, partially covered by the sclera, was inserted behind the posterior Tenon's fascia (Figure 2(a)), with the posterior scleral pole facing anteriorly (Figure 2(b)). Sutures were performed in three layers. The first layer was a continuous suture of the posterior Tenon's fascia with the four rectus muscles sutured on it (Figure 2(c)). The second layer was the suture of the anterior Tenon's fascia performed with interrupted stitches. The third layer was the conjunctiva closed with a continuous suture. If necessary interrupted sutures might be placed on it to secure the implants (Figure 2(d)).

## 3. Results

We enrolled 19 eyes of 19 patients, 9 males, 10 females (mean age 55.4 ± 9.7 years), for a primary evisceration, and 7 patients, 2 males, 5 females, 7 eyes (mean age 60.2 ± 7.6 years), for secondary implantation in superior sulcus eye deformity.

### 3.1. Primary Procedure

All eyes enrolled in the study did not show visual residual function. We performed surgery for the following reasons: 10 eyes were affected by bulbar phthisis and pain; 5 eyes had corneal perforation and endophthalmitis with no bulbar phthisis; 2 eyes had bulbar phthisis after multiple surgical procedures for glaucoma surgery; 2 eyes had leukocoria with a normal bulbus for an aesthetic reason. In those 19 eyes there were inserted 4 implants of 20 mm of diameter and 15 implants of 22 mm of diameter.

We had no postoperative infection and all patients fitted and external prothesis.

### 3.2. Secondary Procedure

All eyes were previously eviscerated with the insertion of an implant. One patient underwent secondary procedure for the extrusion of a previously implanted silastic implant of 14 mm of diameter; it was inserted a 22 mm bioceramic implant. In the other 6 cases, our procedure was performed to increase the orbital cavity volume in order to reduce superior sulcus deformity. The implant diameter changed from 12 to 20 in one case; from 14 to 22 in two cases; from 16 to 22 in two cases; from 18 to 22 in one case.

All patients were followed at least for twelve months (average followup 16.6 months). All patients fitted an external prothesis in the operated eye. We did not find complications, such as implant infection, implant exposure, socket discomfort, or conjunctival thinning.

## 4. Conclusion

Many authors consider evisceration to be a procedure that allows better movement than enucleation [15, 16].

To prevent socket volume loss, superior sulcus depression, and secondary eyelid malposition, it is necessary to place an orbital implant within the sclera with a volume of 4 mL to 5 mL (20–22 mm implant) [17].

Unfortunately, most patients who undergo an evisceration do not have scleral dimension to accommodate an implant larger than 16 mm [11].

This is the reason why the cornea is sometimes retained. Unfortunately, a whitish cornea can be unpleasant to look at and it may cause pain. The use of a small implant does not allow complete fullness of the orbital cavity and the result is an inadequate sustain of the external prothesis. It means that the target of better eye movement with evisceration, in the majority of cases, is not achieved due to an insufficient orbital volume filling.

In our study we showed that it is possible to partially cover porous orbital implant, such as aluminium oxide (Bioceramic), with the patient's sclera. Our procedure starts by performing an enucleation. Once the implant is inserted in the orbital cavity the scleral tissue is put in front of it. Therefore, we obtained a good healing plane for the posterior and anterior Tenon's fascia and conjunctiva. Our procedure allows satisfactory movement of the external prothesis because the four rectus muscles are sutured to the posterior Tenon's fascia and because with this technique the orbital volume is completely filled. Currently, we do not consider the insertion of a peg system in any patient.

In this study, our procedure was safe and effective, with no cases of extrusion, migration, or infection. All patients had a good cosmetic result after final prosthetic fitting.

The bioceramic implant is a safe and effective treatment for blind painful eye. Eyes undergoing evisceration with implantation of this implant achieve good motility and a good cosmetic result.

The position of the implant behind the posterior Tenon's fascia allows a good cicatrisation of the covering tissues, because this posterior Tenon's fascia has never been operated on. Our procedure might be a good alternative especially in case of ocular phthisis, but larger cohort study group are needed.
